# Molecular autopsy

**DOI:** 10.1007/s12471-018-1157-6

**Published:** 2018-09-13

**Authors:** B. van Driel, M. Michels, J. van der Velden

**Affiliations:** 1 Department of Physiology, VU University Medical Center, Amsterdam, The Netherlands; 2 Department of Cardiology, Erasmus Medical Center, Rotterdam, The Netherlands

In this month’s issue of the *Netherlands Heart Journal*, Vos et al. describe the findings of a comprehensive autopsy study into all cases of unexplained and unexpected deaths in minors (0–17 years) in the Netherlands over a period of 15 months [1]. An interesting finding here is that in 25% of cases no structural abnormalities were found at autopsy, which suggests sudden cardiac death (SCD) caused by an inherited arrhythmogenic disorder. Vos et al. and others stress the importance of a systematic autopsy for SCD in minors and argue that cardiogenetic screening, a ‘molecular autopsy’, should be performed when the autopsy does not reveal a structural abnormality which explains the cause of death [2, 3]. What are arguments for and against molecular autopsy after SCD in minors?

Multiple studies have revealed a genetic predisposition to sudden death and that risk for SCD is increased with a family history of SCD [4–8]. Furthermore, a cardiogenetic disorder is found in approximately 50% of families of SCD victims [9–12]. Therefore, systematic autopsy with an optional molecular autopsy after sudden death in minors could lead to the identification of family members at risk for SCD. Pooled results of systematic molecular autopsies can also help to identify markers that improve the accuracy of SCD risk prediction models, which will likely improve SCD prevention strategies when more families with inheritable arrhythmogenic disorders are identified. The prevalences of arrhythmogenic disorders are currently thought to be underestimated, due to incomplete penetrance, the lack of clinical phenotype of these disorders, and incomplete family screening. Increased cardiogenetic screening, not only by molecular autopsy, will bring the estimated prevalences closer to their actual values. Currently only ~40% of family members are screened; from a public health viewpoint, this should be 100% so that SCD may be prevented in those at high risk [13].

However, ethical, legal and financial problems emerge with attempts to increase genetic screening. Genetic counselling by a clinical geneticist is essential to guide family members in the decision-making process towards genetic screening. People have a right not to know, and fear or anticipatory stress play a role in the choice to be screened. Legally speaking, medical confidentiality should be maintained but should be balanced against informing the relatives and respecting their right to be informed. From a financial point of view, payment for genetic testing is an issue because medical insurance stops after death. Furthermore, carrying a disease-causing mutation can hinder the possibility of obtaining life insurance higher than 268,000 €. Considering this, the road to systematic molecular autopsy after SCD in minors is long and winding.
